# Botany, Traditional Use, Phytochemistry, Pharmacology and Quality Control of *Taraxaci herba*: Comprehensive Review

**DOI:** 10.3390/ph17091113

**Published:** 2024-08-23

**Authors:** Jianhao Wu, Jialin Sun, Meiqi Liu, Xiaozhuang Zhang, Lingyang Kong, Lengleng Ma, Shan Jiang, Xiubo Liu, Wei Ma

**Affiliations:** 1 School of Pharmacy, Heilongjiang University of Chinese Medicine, Harbin 150006, China; 17515281505@163.com (J.W.); klp15sjl@nefu.edu.cn (J.S.); mmq660736@163.com (M.L.); zxz999123@163.com (X.Z.); hljkly970219@163.com (L.K.); 18739466784@163.com (L.M.); 17390928032@163.com (S.J.); 2 School of Jiamusi, Heilongjiang University of Chinese Medicine, Jiamusi 154007, China

**Keywords:** *Taraxaci herba*, botany, phytochemistry, pharmacology, quality control, review

## Abstract

*Taraxaci herba*, as a traditional Chinese medicine, is the name of the *Taraxacum* genus in the *Asteraceae* family. Documented in the Tang Herbal Medicine (Tang Dynasty, AD 657–659), its medicinal properties cover a wide range of applications such as acute mastitis, lung abscess, conjunctival congestion, sore throat, damp-heat jaundice, and vision improvement. In the Chinese Pharmacopoeia (Edition 2020), more than 40 kinds of China-patented drugs containing *Taraxaci herba* were recorded. This review explores the evolving scientific understanding of *Taraxaci herba*, covering facets of ethnopharmacology, botany, phytochemistry, pharmacology, artificial cultivation, and quality control. In particular, the chemical constituents and pharmacological research are reviewed. *Taraxaci herba* has been certified as a traditional medicine plant, and its flavonoids, phenolic acids, and terpenoids have been identified and separated, which include Chicoric acid, taraxasterol, Taraxasteryl acetate, Chlorogenic acid, isorhamnetin, and luteolin; they are responsible for anti-inflammatory, antioxidant, antibacterial, anti-tumor, and anti-cancer activities. These findings validate the traditional uses of *Taraxaci herba* and lay the groundwork for further scientific exploration. The sources used in this study include Web of Science, Pubmed, the CNKI site, classic monographs, the Chinese Pharmacopoeia, the Chinese Medicine Dictionary, and doctoral and master’s theses.

## 1. Introduction

*Taraxaci herba* is the name of the *Taraxacum* genus in the *Asteraceae* family, which include *T. mongolicum*, *T. sinicum Kitag*, and several plants of the same genus, and the whole grass is used for medicine. Dandelions are the English common name of *Taraxaci herba*. *Taraxaci herba* is widely distributed in the north temperate zone, including the northeast, north, northwest, central, east, and southwest provinces of China [1,2], and it is also widely distributed in Korea, Mongolia, and Russia, mainly in hillside grasslands, roadsides, fields, and river beaches at middle and low altitudes [3,4].

According to the Chinese Pharmacopoeia (Edition 2020) [5], the medicinal effects of *Taraxaci herba* involve clearing heat and eliminating toxins, hematoma, inflammation, diuresis, and jaundice. *Taraxaci herba* can be employed to treat acute mastitis, lung abscess, conjunctival congestion, sore throat, damp-heat jaundice, and so forth. The dosage of decoction is 10–15 g, and the maximum dosage is 60 g. In addition, the edible part of *Taraxaci herba* is as high as 84%. People in East Asia have the habit of eating Dandelion pickled products, Dandelion porridge, and Dandelion soup since ancient times [6,7].

*Taraxaci herba* is composed of a variety of complex chemical components. The main ones that determine its biological activity are flavonoids, terpenoids, phenolic acids, and polysaccharides. Pharmacological experiments have confirmed that it has anti-inflammatory, antioxidant, antibacterial, anti-cancer, and anti-tumor effects [8,9,10,11]. It has become an urgent matter to clarify the effective components and pharmacological effects of *Taraxaci herba* and find out the factors that affect the accumulation of effective components in *Taraxaci herba* so as to improve the yield and quality of *Taraxaci herba*. In this review, the chemical composition, biological activity, growth conditions, and quality control of *Taraxaci herba* were integrated and analyzed, with the aim of providing a reference for the establishment of a planting system and quality control system of *Taraxaci herba* and the development of new products.

## 2. Materials and Methods

Relevant literature was obtained from scientific databases such as PubMed (https://pubmed.ncbi.nlm.nih.gov, accessed on 6 June 2024), SciFinder, Web of Science, and CNKI (https://www.cnki.net, accessed on 6 June 2024). The keywords used to search were “*Taraxaci herba*”, “Phytochemistry”, “Pharmacology”, “Dandelion”, and “Quality control”. TCMSP (https://old.tcmsp-e.com/tcmsp.php, accessed on 8 June 2024), Pubchem, and Web of Huayuan (https://www.chemsrc.com, accessed on 8 June 2024) were used to find the chemical composition of *Taraxaci herba*. And the chemical structures were accurately depicted using the ChemDraw 23 software. The review also included results from the Flora of China (https://www.iplant.cn/frps, accessed on 10 June 2024), China Plant Science Data Center (https://www.plantplus.cn/cn, accessed on 11 June 2024), Plants of the World Online (https://powo.science.kew.org, accessed on 20 June 2024), classic monographs, Chinese Pharmacopoeia, and doctoral and master’s theses.

## 3. Botanical Features and Distribution

The original plant of *Taraxaci herba* belongs to the genus *Taraxacum*, which mainly grows in temperate and subtropical regions of the northern hemisphere. In China, *Taraxacum* plants are widely distributed in the northeast, north, northwest, central, east, and southwest provinces [1,2]. *Taraxacum* plants contain Taraxasterol, stigmasterol, sitosterol, choline, organic acids, and inulin, so they have very high medicinal and nutritional values [12,13].

**Figure 1:**
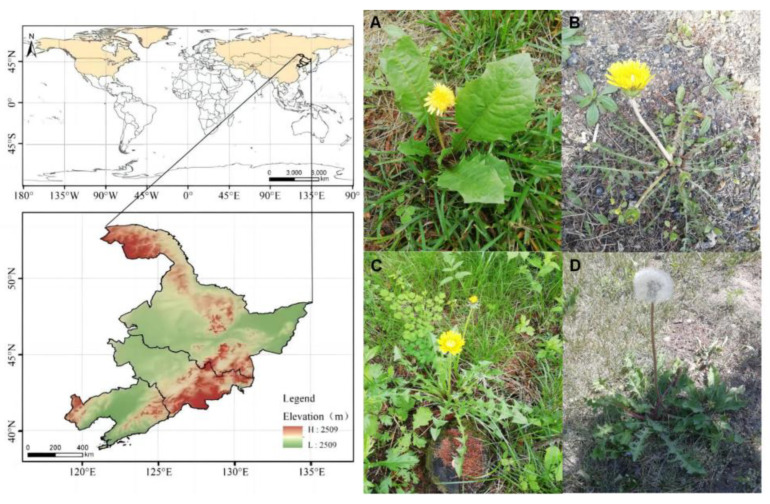
Distribution of *Taraxaci herba* (the yellow shading represents the distribution of *Taraxaci herba*; white is the area where *Taraxaci herba* almost does not exist, and the bottom half of the image is the typical height legend of *Taraxaci herba*): (**A**–**C**) Growth period of *Taraxaci herba*; (**D**) Seed maturity of *Taraxaci herba*.

With reference to China Dictionary of Chinese Herbal Medicine Resources, Flora of China (https://www.iplant.cn/frps, accessed on 1 July 2024), China Plant Science Data Center (https://www.plantplus.cn/cn, accessed on 2 July 2024), and other references [1,2,14,15,16,17], 84 species of *Taraxacum* distributed in China have been identified, as shown in Table 1. And the distribution map of the *Taraxacum* genus is presented in Figure 1.

In China, the most important medicinal species of *Taraxacum* is *T. mongolicum*. *T. mongolicum* is a perennial herb with cylindrical and curved roots, 3–7 cm long, dark brown, and stout. Leaves obovate-lanceolate, obovate-lanceolate, or oblong-lanceolate, 4–20 cm long and 1–5 cm wide, with blunt or acute apex and sometimes wavy teeth or pinnately parted edges, with 3–5 lobes on each side, triangular or triangular-lanceolate, usually toothed; Scapes 1 to several, equal to or slightly longer than leaves, 10–25 cm high; The capitulum is about 30–40 mm in diameter; Involucre bell-shaped, 12–14 mm long, pale green, involucre 2–3 layers [18]. The tongue-shaped flower is yellow, with a tongue length of about 8 mm and a width of about 1.5 mm. The back of the edge flower tongue has purple-red stripes, and the anther and stigma are dark green. The flowering period is 4–9 months [19]. *T. sinicum* is also one of the medicinal varieties of *Taraxacum*. Scapes 1 to several, 5–20 cm high, longer than leaves; The capitulum is about 20–25 mm in diameter; The involucre is small, 8–12 mm long, light green, and the involucre has three layers; Tongue-shaped flowers are yellow and thin white. The tongue is about 8 mm long and 1–1.5 mm wide, and the flowering period is 6–8 months. Achenes obovate-lanceolate, light brown, about 3–4 mm long; The crest is white and 5–6 mm long [18,20]. In addition, *T. scariosum*, *T. platycarpum*, *T. officinale*, and other plants of *Taraxacum* are often used as medicinal varieties.

## 4. Traditional Uses

*Taraxaci herba* is called Pugongying (Dandelion) in China’s classical medical works. *Taraxaci herba* has a long medicinal history and was recorded as a medicinal plant for the first time in Liu Juanzi Gui Yifang (Jin Dynasty, AD 442–499), and it was recorded that *Taraxaci herba* soup can cure “Carbuncle of breast”. Tang Bencao (Tang Dynasty, AD 657–659) introduced the medicinal value of *Taraxaci herba* systematically to the government for the first time. Bencao Yanyi (Song Dynasty, AD 1116) and Bencao Yanyi Buyi (Yuan Dynasty, AD 1347) systematically introduced *Taraxaci herba* from the unique Four natures of drugs, Five flavors and Channel tropism of traditional Chinese medicine. In addition, Bencao Gangmu (Ming Dynasty, AD 1578–1596), Diannan Bencao (Ming Dynasty, AD 1436), and Gangmu Shiyi (Qing Dynasty, AD 1765–1864) also supplemented the medicinal functions of *Taraxaci herba*. In addition, the medical classics of ethnic minorities in China, such as Tibetan Medicine in China, Qingzang Yaojian, Mengzhi Yaozhi, Dianyao Lu, Miao Yiyao, Laoai, Tujia Yao, and Dedai Yao, also contain relevant records about *Taraxaci herba* as a medicinal plant.

*Taraxaci herba* is widely used in traditional Chinese medicine prescriptions, such as Yingtengtang, Zhirubianyongfang, Lixiaotang, Pugongsan, and Kaiyaguchiqifang, which all take *Taraxaci herba* as the main ingredient, and have been widely used in clinics. At present, there have been reports regarding the combination of *Taraxaci herba* with other traditional Chinese medicines in the treatment of mastitis [21,22], colitis [23], acute tonsillitis [24], and intestinal obstruction [25]. This article collects a large number of traditional Chinese medicine formulas about *Taraxaci herba*, as shown in Table 2.

## 5. Phytochemistry

*Taraxaci herba* possesses distinctive biological characteristics and excellent biological activity, and its complex chemical composition determines its unique biological activity. The main chemical components of *Taraxaci herba* include flavonoids, phenolic acids, polysaccharides, terpenoids, volatile oils, and alkaloids, among which flavonoids, phenolic acids, and terpenoids are the main active components of *Taraxaci herba*.

### 5.1. Flavonoid

Flavonoids are a type of natural product that widely exists in the plant kingdom and generally take C6-C3-C6 as the basic carbon chain skeleton. Flavonoid metabolites generally possess the ability of defense, allelopathy, inhibition of harmful bacteria, and antioxidation [38]. The grass, roots, stems, leaves, and flowers of *Taraxaci herba* all contain flavonoids, and the aboveground parts are the most abundant. The content of flavonoids in *Taraxaci herba* is approximately 1.35%, of which luteolin (**1**) is the highest, followed by luteolin-7-O-β-D-glucoside [39,40]. Yun Ling separated 9 kinds of flavonoids from the ethanol crude extract of *Taraxaci herba*, which were as follows luteolin (**1**), diosmetin [41], apigenin (**11**), hesperetin 7-O-β-D-glucuronide (**19**), rutin [41], isoquercitrin (**17**), quercetin-3-O-β-D-galacoside, quercetin (**2**), luteolin-7-O-β-D-glucoside [39,42,43]. Yao Wei [44] also separated luteolin (**1**), luteolin-7-O-β-D-glucoside [39], and quercetin (**2**) from the crude ethanol extract of *Taraxaci herba* by chromatography. Rong Wang et al. [45] extracted hesperetin 7-O-β-D-glucocuronide (**19**) and hesperetin 5-O-glucoside (**20**) from *Taraxaci herba* for the first time by optimizing the water extraction method. Milovanovic Stoja extracted the flavonoids (208.6–564.5 μg QE/g) and phenolic (5.5–12.1 mg GAE/g) active components from dandelion seeds by supercritical fluid extraction technology [46].

**Figure 2:**
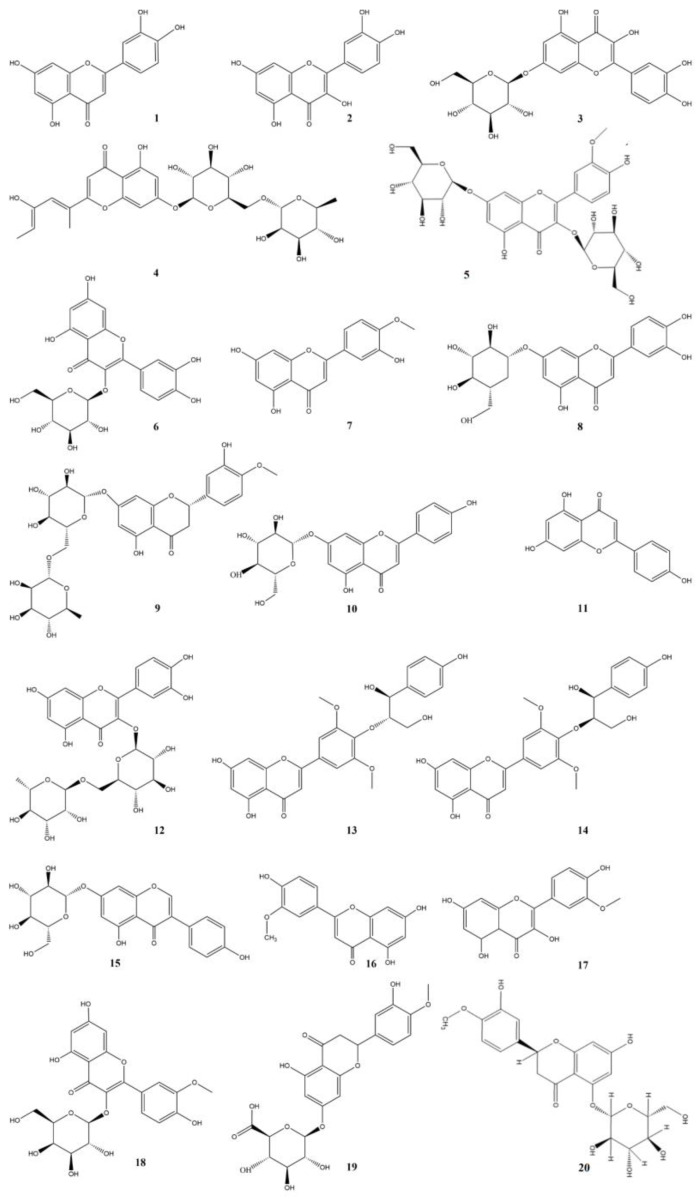
Structures of flavonoids isolated from the *Taraxaci herba*.

Flavonoids, due to their remarkable functions such as liver protection, bacteria inhibition, and oxidation resistance, are widely utilized in the health care products, medicine, and cosmetics industries. Hence, we have summarized the previous research reports regarding flavonoids in *Taraxaci herba*, as presented in Table 3 and Figure 2.

### 5.2. Terpenoids and Steroids

Terpenoids are the products of advanced plant evolution and are the general names of isoprene polymers and their derivatives, which are usually expressed by the general formula of carbon skeleton (C_5_H_8_)_n_ [52]. Triterpenoids and sesquiterpenes are the main terpenoids in *Taraxaci herba*, which possess anti-inflammatory and anti-tumor activities [53].

The triterpenoids in *Taraxaci herba* are mainly pentacyclic triterpenoids. Yun Ling [54] initially discovered two triterpenoids from the crude ethanol extract of *Taraxaci herba*, namely psi-taraxasterol acetate (**1**) and psi-taraxasterol (**4**) palmitate. Taraxasterol [41] is a triterpene compound isolated and identified from the pollen, roots, and leaves of *Taraxaci herba*, and it is one of the main active components of *Taraxaci herba* with anti-inflammatory and anti-tumor effects. Schütz et al. isolated taraxasterol [41], psi-taraxasterol (**4**), and arnidiol [41] from *Taraxaci herba*, and the content in the root of *Taraxaci herba* was significantly higher than that in other parts [9]. Sesquiterpenes in *Taraxaci herba* are mainly sesquiterpene lactones (**19**), and sesquiterpenes are the main components of the bitter source of *Taraxaci herba* [55]. Zhiyun Meng et al. identified the content of monomeric sterols in *Taraxaci herba*. The results showed that the content of β-sitosterol (**11**) was the highest, followed by stigmasterol (**13**) and campesterol (**15**) [56].

**Figure 3:**
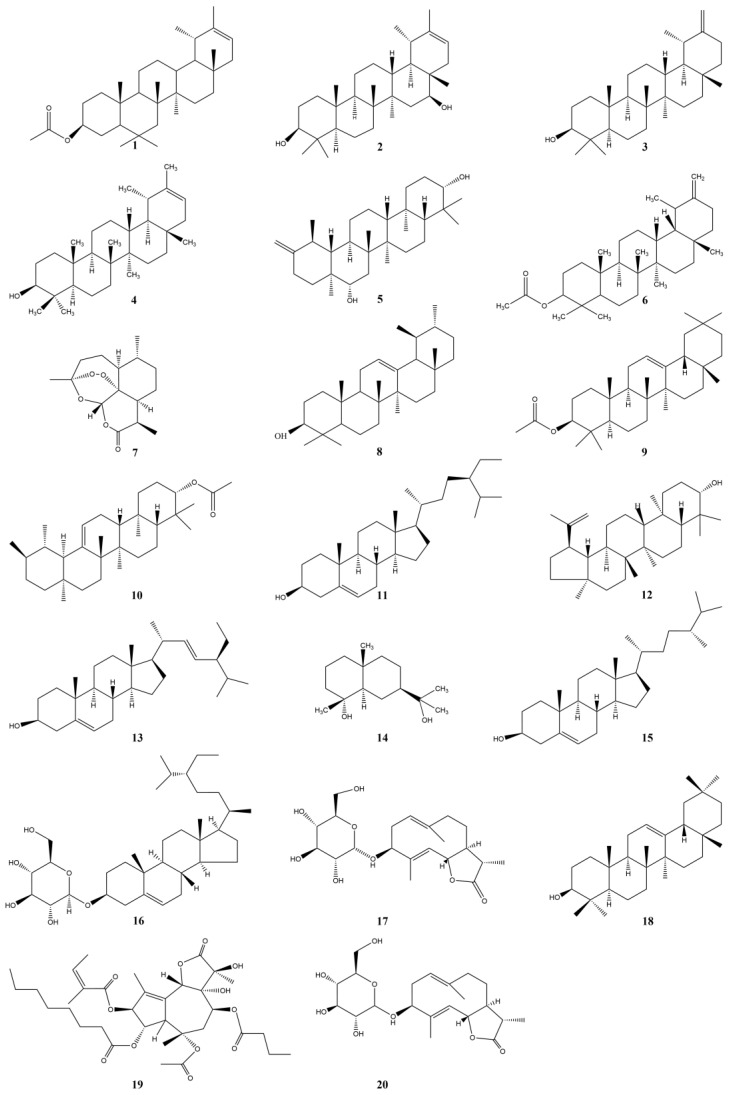
Structures of Terpenoids and Steroids isolated from the *Taraxaci herba*.

In order to present the terpenoids in *Taraxaci herba* more intuitively, we choose to present them in tabular form, as shown in Table 4 and Figure 3.

### 5.3. Phenolic Acid

Phenolic acid compounds are one of the important secondary metabolites in *Taraxaci herba*, which are a kind of compounds formed by combining one or more aromatic rings with one or more hydroxyl groups, and have certain anti-inflammatory, antibacterial, anti-cancer, and antioxidant activities [64].

Shuyun Shi has successfully extracted phenolic acids such as gallic acid (**11**), ferulic acid [41], chlorogenic acid (**1**), caftaric acid [41], and caffeic acid [41] from *Taraxaci herba* [61,65,66,67]. Deqian Peng et al. isolated three phenolic acids from *Taraxaci herba* roots for the first time, namely ethyl (p-Hydroxyphenyl) acetate, methyl caffeate acid (**13**), and vanillin (**17**) [68]. Kenny O et al. isolated and identified three kinds of phenolic acids with antibacterial activity from the methanol crude extract of the root of *Taraxaci herba*, including vanillin (**17**), coniferaldehyde (**18**), and p-methoxyphenylglyoxylic acid (**19**) [57].

**Figure 4:**
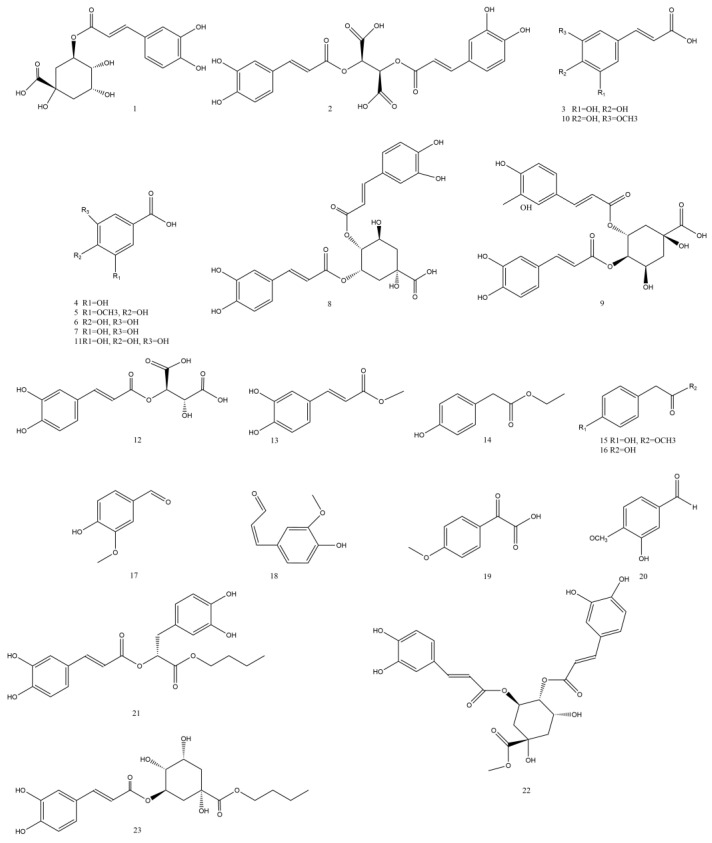
Structures of phenolic acids isolated from the *Taraxaci herba*.

Currently, the phenolic acids isolated and identified from *Taraxaci herba* are presented in Table 5 and Figure 4.

### 5.4. Others

*Taraxaci herba* is abundant in polysaccharides, pigments, alkaloids, volatile oils, and other chemical components [71,72,73]. Hongkun Xue extracted polysaccharides by an ultrasonic-assisted hot water extraction method. The highest polysaccharide yield was (12.08 ± 0.14)%, and the IC50 values for scavenging DPPH and OH free radicals were 0.71 mg/mL and 0.75 mg/mL, respectively [74]. The polysaccharide in *Taraxaci herba* accounts for 30~50% of its dry weight, and the polysaccharide content in *Taraxaci herba* root is 83.31%, which is significantly higher than that in leaves and flowers, and the inulin content accounts for 45% of the root [75]. *Taraxaci herba* contains certain fatty acids, such as myristic acid, linoleic acid, palmitic acid, and linolenic acid, as well as various amino acids, proteins, and minerals [76,77].

## 6. Pharmacology

### 6.1. Anti-Inflammatory and Liver Protection

In recent years, the anti-inflammatory effect and mechanism of pure compounds and crude extracts of *Taraxaci herba* plants have been studied through cell and animal models, which have proven that the anti-inflammatory effect of *Taraxaci herba* plants is remarkable. Taraxasterol (TS) is one of the main active components in *Taraxaci herba*, which plays an anti-inflammatory role, and its content is the highest in the roots [78,79]. Xuemei Zhang et al. utilized the lipopolysaccharide (LPS) induced inflammatory model of peritoneal macrophages in mice to assess the anti-inflammatory effect of TS. The experimental results indicate that TS can inhibit the production of inflammatory factors PGE2, TNF-α, IL-1, and IL-6 in inflammatory tissue cells and prevent NF-κB from translocation from cytoplasm to nucleus, thereby exerting an anti-inflammatory role [80]. San Zhihao perfused the mouse mammary duct with LPS, thus establishing a mouse mammary inflammatory injury model to evaluate the anti-inflammatory activity of TS [81]. The results demonstrated that the production of inflammatory factors TNF-α, IL-1β, and IL-6 in breast tissue was inhibited, the activation of NF-κB signaling pathway was blocked, and MPO activity decreased, suggesting that TS has obvious anti-inflammatory activity. Feng Zheng et al. used LPS to stimulate human umbilical vein endothelial cells (HUVECs) to establish a vascular inflammation model and explored the anti-inflammatory effect and mechanism of TS. The experimental results show that the anti-inflammatory mechanism of TS inhibits the production of inflammatory factors TNF-α, IL-8, IL-1β, and NO and suppresses the activation of NF-κB and the expression of VCAM-1 and ICAM-1 [82]. By observing the effect of TS on hepatocytes of mice with LPS-induced hepatitis, Yifang Yi et al. discovered that TS regulates the JAK2/STAT3 and JNK signaling pathways by up-regulating the expression of the SOCS3 gene, thus inhibiting the inflammatory reaction of mouse hepatocytes [83]. Bingjie Ge et al. used acetaminophen (APAP) to stimulate AML12 cells to establish a mouse liver injury model in order to clarify the protective mechanism of TS on liver injury. The results showed that TS could alleviate the pathological changes of the AML12 cells, promote the expression of the Nrf2/HO-1 pathway, inhibit JNK phosphorylation, reduce the ratio of Bax/Bcl-2 and the expression of caspase-3, demonstrating unique anti-inflammatory and liver-protecting activities [84].

*Taraxaci herba* polysaccharides (TPS), as another potential anti-inflammatory component in *Taraxaci herba*, have been increasingly reported in relation to their anti-inflammatory mechanism. Park, through the influence of TPS on LPS-stimulated RAW 264.7 cells, explores the anti-inflammatory mechanism of TPS. Studies have shown that TPS can effectively inhibit the inflammatory reaction mediated by NFκB by regulating the PI3K/Akt pathway and stimulate the antioxidant potential mediated by Nrf2 [85]. Chaoyong Xiao explores the anti-inflammatory mechanism of TPS through the LPS-induced RAW264.7 cell model. The experiment showed that TPS significantly inhibited the mRNA expression of inflammatory factors COX-2, TNF-α, IL-6, and IL-1β in the cell model, which provided the basis for its anti-inflammatory activity [86]. Taking the LPS-induced enteritis model as the research object, Zhu Li et al. explored the protective and therapeutic effects of TPS on acute enteritis. The results show that TDS can not only promote the repair of the intestinal barrier in the enteritis model but also down-regulate the expression of inflammatory factors (TNF-α, IL-6, IL-1β) and up-regulate the expression of the anti-inflammatory factor IL-22, thereby protecting acute intestinal inflammation [75]. In addition, Han Seok Hee found that the ethanol extract of *Taraxaci herba* significantly inhibited the production of NO and inflammatory factors (Cox-2, TNF-α, IL-6, IL-1β), thus effectively alleviating intestinal inflammation [87]. To sum up, TS and TDS in *Taraxaci herba* are the main active components that play an anti-inflammatory role, and they can reduce the inflammatory damage of the host by inhibiting the secretion of inflammatory factors. The above modern pharmacological and animal experimental studies provide a theoretical basis for dandelion as a traditional anti-inflammatory medicine plant, and the anti-inflammatory mechanism of *Taraxaci herba* should be further explored for the development and utilization of new drugs. Jun Qi et al. have achieved remarkable results in treating acute mastitis with *Taraxaci herba* in the clinic. Among the 58 patients in the treatment group, 57 of them were treated with *Taraxaci herba*, and the redness, swelling, fever, and pain were relieved or disappeared, while the body temperature was significantly decreased or returned to normal, but one case was ineffective [21]. Nan Qiao used *Taraxaci herba* combined with minimally invasive catheter drainage to treat 89 patients with acute mastitis, and a control group was set up. Compared with other groups, the combined treatment group has the advantages of less trauma, quicker recovery, and no impact on breastfeeding [22]. Doctor Lijing An treated patients with acute mastitis using *Taraxaci herba* combined with cefradine, and an experimental group and a control group were respectively set up. The experimental group was the combined medication group, while the control group was given cefradine intravenously. After treatment, the levels of inflammatory factors IL-6, PCT, ICAM-1 and TNF-α in both groups groups decreased significantly, and the experimental group was superior to the control group [88].

### 6.2. Anti-Oxidation

Flavonoids are the main antioxidant products of *Taraxaci herba*, which have an obvious anti-aging effect [89]. Mingyue Pan extracted flavonoids from *Taraxaci herba* by means of ethyl acetate and dichloromethane and tested their antioxidant activity. The results showed that *Taraxaci herba* flavone (TF) had a strong scavenging ability for hydroxyl radical (OH), superoxide anion (O2), and 1,1-diphenyl-2-picrylhydrazyl (DPPH), and its main component was isorhamnetin [90]. The flavonoids extracted from the flowers of *Taraxaci herba* have a scavenging ability for superoxide anion (O2) and hydroxyl radical (OH) and cooperate with α-tocopherol to scavenge DPPH to play an antioxidant role [91]. In addition, the antioxidant activity of TF is related to the up-regulation of the mRNA levels of Nrf2 and SOD1, which leads to the increase of SOD and GSH and the decrease of MDA [92].

Not only does TF play an antioxidant role, the polysaccharides in *Taraxaci herba* also have obvious antioxidant activity. By extracting TDS with water and analyzing its antioxidant activity, Yunfeng et al. found that TDS can effectively neutralize DPPH, superoxide anion (O2), and hydroxyl radical (OH), suggesting that it has obvious antioxidant activity [93]. Zhou studied the antioxidant mechanism of TDS through animal model experiments. The results show that TDS can exert antioxidant activity by reducing the level of malondialdehyde (MDA) and increasing the activities of superoxide dismutase and glutathione peroxidase [94]. TDS activates a series of antioxidant genes via Nrf2 and then up-regulates the activity of antioxidant protease to play an antioxidant role [95,96]. To sum up, *Taraxaci herba* can reduce the oxidative damage of cells by scavenging free radicals and improving the activity of antioxidant enzymes, which provides convenience for developing *Taraxaci herba* as a new antioxidant.

### 6.3. Anti-Bacterial Activity

*Taraxaci herba* has a broad anti-bacterial spectrum and obvious anti-bacterial activity. Through the anti-bacterial activity verification of the ethanol extract of Taraxaci herba, Demin verified that it has obvious antibacterial activity against staphylococcus aureus and escherichia coli [97]. Qiao and Sun respectively tested the antibacterial ability of the ethanol and ethyl acetate extracts from the flowers of *Taraxaci herba* and determined the MIC value. The results showed that the anti-bacterial activity of the ethyl acetate extract against Pseudomonas aeruginosa and Bacillus subtilis (the MIC values were 125 μg/mL and 62.5 μg/mL, respectively) was significantly stronger than that of the ethanol extract [98]. Katy tested the antibacterial activity of the ethyl acetate extract from the leaves of *Taraxaci herba*. The results showed that the extract of *Taraxaci herba* leaves had relatively strong antibacterial activity against staphylococcus aureus, with a MIC of 200 μg/mL, and the MIC of escherichia coli and streptococcus pneumoniae was 400 μg/mL [99]. Xuefeng found that the phenolic extract from the *Taraxaci herba* flower can rupture the cell membrane of staphylococcus aureus, cause the leakage of adenosine triphosphate and Na-K-ATPase in the cell, and lead to bacterial death. Its main active components are Caffeic acid and Luteolin-7-O-glucoside [100].

**Figure 5:**
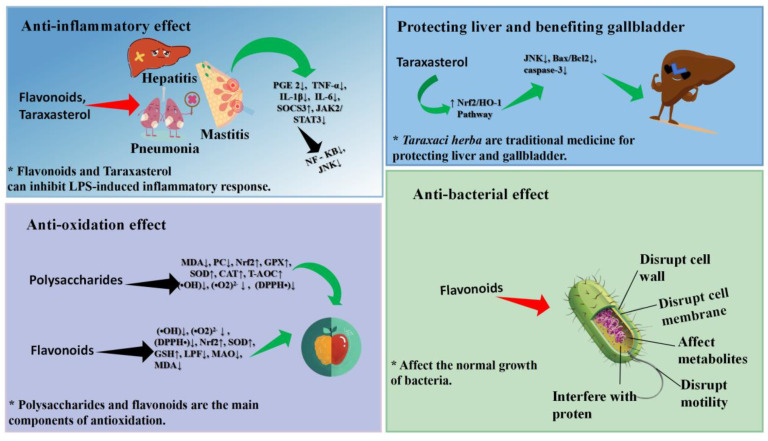
The Pharmacological properties of *Taraxaci herba*.

In addition, *Taraxaci herba* also possesses certain antiviral and antifungal pharmacological activities. Yinku et al. found that the ethanol extract of *Taraxaci herba* could destroy the cell wall and cell membrane of candida albicans, resulting in the leakage of nucleic acid macromolecules, destroying cell metabolism and ultimately leading to the death of candida albicans [101]. At the concentration range of 25~100 μg/mL, the combined extract of *Taraxaci herba* ethanol and ethyl acetate significantly blocked the protein synthesis and DNA replication of the hepatitis B virus, and the antiviral effect was remarkable [102]. The aqueous extract of *Taraxaci herba* has potential anti-human immunodeficiency virus (HIV) activity, and its mechanism may be the inhibition of HIV-1 replication and reverse transcriptase activity [103]. In addition, *Taraxaci herba* also has an anti-hepatitis C virus (HCV) effect [104]. That is to say, the *Taraxaci herba* extract exerts anti-bacterial activity by destroying cell walls and cell membranes and interferes with the replication of virus DNA and protein, thus exerting antiviral activity. The drug concentration shows a positive correlation with both the rate of inhibition of bacterial growth and the rate of inhibition of biofilm formation. We present the pharmacological effects of *Taraxaci herba* in the form of pictures. For details, please refer to Figure 5.

### 6.4. Anti-Tumor and Anti-Cancer

Cancer is the most prevalent type of malignant tumor and one of the main causes of human mortality. In recent years, its incidence rate has been on the rise. Based on clinical practice and modern pharmacological research, *Taraxaci herba* has remarkable anti-tumor effects, mainly in breast cancer, liver cancer, stomach cancer, bladder cancer, and so on [105,106].

#### 6.4.1. Breast Cancer

Breast cancer is a heterogeneous disease with significant individual differences. It ranks first among the most common cancers in women and has a high mortality rate [107,108]. Sophia conducted a study on the anti-cancer mechanism of *Taraxaci herba* for the first time. The aqueous extract of *Taraxaci herba* leaves inhibited the proliferation and differentiation of breast cancer cells (MCF-7) in an ERK-dependent manner, which exhibited a certain anti-cancer effect [109]. By observing the apoptosis of TNBC cell line under the influence of the ethanol extract of *Taraxaci herba*, Li et al. studied the anti-cancer mechanism of *Taraxaci herba*. The experimental results indicated that the ethanol extract of *Taraxaci herba* significantly reduced the activity of MDA-MB-231 cells, triggered G2/M phase arrest, increased caspase-3 and PARP protein, and promoted the apoptosis of cancer cells [110]. Shan combined network pharmacology and multiomics technology to study the mechanism of action of the ethanol extract of *Taraxaci herba* on breast cancer. The results showed that the anti-cancer mechanism of the ethanol extract of *Taraxaci herba* might be down-regulating the expression of CHKA, inhibiting the PI3K/AKT pathway and its downstream targets SREBP and FADS2 to interfere with the metabolism of glycerophospholipid and unsaturated fatty acids [111]. According to Xin-Xin et al., the experimental results show that the ethanol extract of Taraxaci herba can inhibit the proliferation, migration, and invasion of TNBC cells in the TAMs microenvironment by inhibiting the IL-10/STAT3/PD-L1 signaling pathway [112].

Hu Niu et al. extracted TDS by ultrasonic extraction and constructed a tumor model of breast cancer MCF-7 cells transplanted subcutaneously in nude mice to study the anti-tumor activity of TDS in vivo. The experimental data showed that TDS could significantly increase the expression of Pro-apoptosis proteins (p53, Bax) and significantly decrease the expression of anti-apoptosis protein Bcl-2 in MCF-7 cells, which was positively correlated with the dose [113]. Yumin observed the effects of different concentrations of Taraxaci herba flavonoids (TF) on the proliferation and apoptosis of MCF-7 cells by culturing MCF-7 cells in vitro. The results showed that TF promoted the apoptosis of MCF-7 cells by down-regulating the expression of Bcl-2 mRNA and up-regulating the expression of P53 and Bax mRNA [114]. Hamed et al. observed the proliferation and metastasis activities of MCF-7 and MDA-MB231 breast cancer cells under the combined action of TF and all-trans-retinoic acid (ATRA). The results showed that the expressions of MMP-9 and IL-1β in the treated two cell lines decreased significantly, while the expressions of p53 and KAI1 increased, and TF had obvious anti-tumor activity [115]. In addition, the triterpenoid compound TS in *Taraxaci herba* also has obvious Anti-tumor activity, and its mechanism may be that it inhibits the migration and invasion of MDA-MB-231 cells through ERK/Slug axis [116].

#### 6.4.2. Prostate Cancer

Prostate cancer (PCa) accounts for 13.5% of the incidence of male malignant tumors, ranking second. More than 80% of the patients are men over 65 years old, and the incidence rate of urban men is 3.7 times that of rural men [117].

Christopher Nguyen et al., by observing the apoptosis of the PCa cell line induced by water extract of *Taraxaci herba* root, discovered that the *Taraxaci herba* root extract induced PCa cell apoptosis in a dose- and time-dependent manner and also had good tolerance, but its mechanism was not clarified [118]. Therefore, Jingqiu et al., through the modeling experiment of xenotransplantation of the PCa cell line, clarified the mechanism of TS inhibiting the proliferation of prostate cancer cells. The results showed that TS inhibited the proliferation of prostate cancer cells by inhibiting the activation of the PI3K/AKT signaling pathway and the expression of FGFR2 [119]. Morteza, through pharmacological experiments, found that the expression of uPA-uPAR, MMP-9, and MMP-2 in PCa cells treated with TS decreased significantly, while the expression of TIMP-2 and TIMP-1 increased significantly, which confirmed that TS had a therapeutic effect on prostate cancer [120].

#### 6.4.3. Liver Cancer

Hepatocellular carcinoma (HCC) is a malignant tumor of the digestive tract with high morbidity and mortality worldwide, and its treatment and prognosis are difficult [121]. Ren investigated the mechanism of TDS in the treatment of liver cancer (HCC) through cell and animal experiments. The experimental results indicated that TDS significantly increased the spleen index, regulated T cell activation, and inhibited the growth of cancer cells, and the expression levels of P-PI3K, P-AKT, and P-mTOR genes in HCC cells were significantly reduced [122,123].

As one of the components with anti-cancer activity in *Taraxaci herba*, TS also has a remarkable inhibitory effect on liver cancer cells. Feng studied the anti-cancer mechanism of TS through the H22 hepatoma mouse cell model. The results showed that TS could significantly inhibit the proliferation of HCC cells, induce their apoptosis in vitro, and inhibit the growth of cancer cells and the expression of Ki67 in H22 hepatoma mice. The mechanism might be related to the regulation of Apoptosis-related proteins and the IL-6/STAT3 pathway [124]. Tianhao found that the apoptosis of mouse subcutaneous transplanted liver cancer cells induced by TS might be caused by increasing the level of Hint1 to regulate Bax and down-regulating the expressions of Bcl2 and cyclinD1 [41].

#### 6.4.4. Gastric Cancer

Gastric cancer (GC) is the fifth most common cancer and the fourth most common cause of cancer death worldwide, and it is a disease with high molecular and phenotypic heterogeneity [125].

Hui conducted in vitro experiments to test the cytotoxicity of the water extract of *Taraxaci herba* on the human gastric cancer cell line (SGC-7901). The results revealed that the aqueous extract of *Taraxaci herba* could promote the apoptosis of gastric cancer cells, reduce the migration ability of gastric cancer cells, and reduce the expression of pro-proliferation and anti-apoptosis genes (Erk, survivin and Bcl2) [126]. Long-chain noncoding RNA (lncRNA) can impact the proliferation of tumor cells. Through the study of targeting lncRNA-CCAT1 with the aqueous extract of *Taraxaci herba* root, Zhu discovered that CCAT1 in GC cells was significantly down-regulated, and it inhibited the proliferation and migration of GC cells [127]. Wei C established a subcutaneous xenotransplantation model of gastric cancer by subcutaneously injecting MKN-28 cells in nude mice and then observed the growth of gastric cancer cells after TS administration. The results showed that TS inhibited the growth of gastric cancer by down-regulating the expression of EGFR and AKT1 in cancer cells and inhibiting the EGFR/AKT1 signaling pathway [128]. Yang, through cell experiments, it was found that TS inhibited the proliferation of gastric cancer cells and promoted the apoptosis of gastric cancer cells by inhibiting glycolysis mediated by GPD2 [129].

#### 6.4.5. Others Cancer

**Figure 6:**
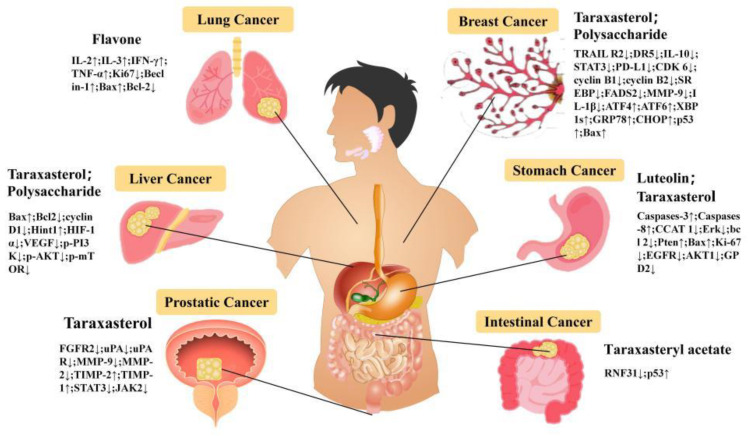
The Anti-Cancer of *Taraxaci herba*.

Colorectal cancer (CRC) is the third most common cancer in the world, as recognized by the WHO [130]. Tao C observed that the mortality of CRC patients with high expression of the RNF31 protein was much higher than that of patients with low expression of RNF31. Taraxasterol acetate could promote the degradation of RNF31 and then inhibit the mutation of the P35 gene, thus playing an anti-cancer role [131,132]. Le, by testing the effect of *Taraxaci herba* flavone (TF) on cancer cells in the Lewis mouse lung cancer model, it was found that under the influence of 200 μg/mL TF, the levels of CD4+, CD8+ and CD4+/CD8+ in cancer cells increased, the levels of IL-2, IL-3, IFN-γ, and TNF-α increased, and the expression of Ki67 decreased significantly, thus inhibiting the proliferation of lung cancer cells and improving the host’s immunity [133]. In addition, *Taraxaci herba* has an inhibitory effect on glioma, leukemia, pancreatic cancer, melanoma, and esophageal cancer in vitro and in vivo, or it can be used as a radiosensitizer to assist in the treatment of cancer [134,135,136]. The anti-cancer and anti-tumor mechanisms of *Taraxaci herba* need to be further explored through experimental research and clinical practice in order to develop them into potential natural anti-cancer drugs. We present the anti-cancer mechanism of *Taraxaci herba* in the form of pictures. For details, please refer to Figure 6.

### 6.5. Other Pharmacological Effects

*Taraxaci herba* not only has certain anti-inflammatory, anti-oxidation, and anti-cancer effects but also has the functions of improving immunity [137,138,139], lowering blood sugar and blood fat [140,141,142,143,144], protecting liver and gallbladder [145,146], diuresis, anticoagulation [147,148] and depression [149].

## 7. Artificial Cultivations

### 7.1. Sowing and Harvesting

*Taraxaci herba* can be sown from April to September every year, which has strong adaptability and can survive in most soils. The seeds of *Taraxaci herba* have no dormancy characteristics, and their vigor decreases rapidly after harvesting. It is best to choose new seeds for sowing, which can generally emerge in 7–15 days, and weeds need to be removed as soon as possible.

The Pharmacopoeia of China (2020 edition) stipulates that *Taraxaci herba* should be harvested at the beginning of flowering from spring to autumn. In order to ensure multiple harvesting, only the aerial parts of *Taraxaci herba* are collected the first few times. The aerial parts of artificially cultivated *Taraxaci herba* can usually be harvested after growing for 2–3 months in the first year and can be used as medicine after removing impurities, washing, and drying in the sun. Traditional methods of drying or refrigerating medicinal materials have damaged the bioavailability of bioactive components in medicinal materials to some extent. In the future, the emerging nano-encapsulation technology will be used to improve the bioavailability of bioactive components [150]. However, wild *Taraxaci herba* usually needs to grow for about 6 months due to insufficient nutritional conditions [151]. The underground part of medicinal *Taraxaci herba* is usually harvested in autumn. Shiyu Li and Minghao Shen determined the content of active ingredients in *Taraxaci herba* roots collected in different periods [152]. The content of flavonoids and saponins in *Taraxaci herba* roots collected at the end of August was the highest (12.78 mg/g, 0.78 mg/g), the choline content in *Taraxaci herba* roots collected at the end of July reached the peak of 5.06 mg/g, and the antibacterial activity of *Taraxaci herba* collected from August to September was the most obvious. Ruiyi et al. The content of active ingredients in *Taraxaci herba* roots at different harvest times was determined [153]. The results showed that the content of flavonoids in *Taraxaci herba* roots reached the peak of 10.61 mg/g at the beginning of September, the content of total polysaccharides reached the peak of 131.98 mg/g at the end of September, and the content of phenolic acids (caftaric acid, chlorogenic acid, caffeic acid, and cichoric acid) reached the highest of 0.98% in mid-September.

### 7.2. Cultivation

*Taraxaci herba*, as a commonly used bulk medicine, should not only ensure the supply of raw materials for pharmaceutical enterprises but also ensure that its medicinal value reaches the standard. Wild species are unsustainable, so it is particularly important to cultivate *Taraxaci herba* artificially. There are many factors that affect the growth of *Taraxaci herba* and the accumulation of its active components, including light intensity, fertilizer dosage, soil environment, harvesting and storage, etc. Therefore, the previous studies are summarized, as shown in Table 6.

## 8. Quality Control and Toxicology

### 8.1. Quality Control

Due to the imperfection of market supervision and the identification system of traditional Chinese medicine, there have always been problems with variety misidentification and adulteration, but as a drug, the quality standard must comply with relevant laws and regulations. At present, the China Pharmacopoeia (2020 edition) [5] regulates the quality of *Taraxaci herba* from three aspects: Character identification, microscopic identification, and TLC. The moisture content of *Taraxaci herba* is no more than 13.0%, the total ash content is no more than 20.0%, and the leaching solution content should not be less than 18.0%. Cichoric acid in *Taraxaci herba* was determined to be ≥0.45% by HPLC, and cichoric acid in processed *Taraxaci herba* pieces was ≥0.30%. The European Pharmacopoeia (11.0 edition) regulates the quality of *Taraxaci herba* from three aspects: Character identification, microscopic identification, and TLC. The loss on drying of *Taraxaci herba* is not more than 12.0%, the total ash content is not more than 10.0%, the proportion of ash insoluble in hydrochloric acid should not exceed 3%, and the leaching solution content should not be less than 20.0% [163]. However, it may not be enough to evaluate the quality system of *Taraxaci herba* only by HPLC. With the progress of science and technology, people can jointly use other methods to determine the substance content of *Taraxaci herba* and improve the quality control system of *Taraxaci herba*.

Chemical fingerprint, the fingerprint of traditional Chinese medicine, is a comprehensive and quantifiable identification method based on the systematic study of chemical components of traditional Chinese medicine, which is used to evaluate the authenticity, stability, consistency, and effectiveness of traditional Chinese medicine [164,165]. Aipeng L established a new chemical fingerprint of *Taraxaci herba*, corrected nine common peaks, and determined the contents of chlorogenic acid, cichoric acid, caffeic acid, caftaric acid, and Isochlorogenic acid A and luteolin can be used as markers to evaluate the quality of *Taraxaci herba* [166]. Bo H, the fingerprints of 15 batches of *Taraxaci herba* from different habitats were determined, 23 common peaks were identified, and the contents of 11 chemical components (chlorogenic acid, chicoric acid, caffeic acid, quercetin, apigenin, rutin, diosmetin, luteolin-7-O-β-D-glucoside, kaempferol, genkwanin, 7-Hydroxycoumarine) were identified [167]. Meng Ran et al. calibrated six common peaks by fingerprint combined with multi-component content determination and chemical pattern recognition and determined that caftaric acid, chlorogenic acid, caffeic acid, and cichoric acid can be used as material indexes for quality evaluation of *Taraxaci herba* [168]. Twenty-two phenolic compounds in *Taraxaci herba* were determined by HPLC-DAD-MS/MS, and cichoric acid, caffeic acid, and luteolin were identified as quality markers, which further improved the quality evaluation system of *Taraxaci herba* [169]. Wu Zhe, through the comparative analysis of chemical fingerprint and high-performance liquid chromatography, caftaric acid, chlorogenic acid, caffeic acid, and cichoric acid were selected as quality evaluation markers of *Taraxaci herba* [64]. With the continuous development of technology, the quality control methods of medicinal materials are also constantly innovating, and the innovation of quality control methods and technologies of *Taraxaci herba* is becoming more and more important.

### 8.2. Toxicology

To date, little literature has reported the toxicity of *Taraxaci herba*. People’s Republic of China Pharmacopoeia (2020 edition) [5] stipulates that the dosage of dried dandelion is 10–15 g, and no adverse reactions have been reported in normal dosage. Up to now, only 3 cases of adverse reactions caused by clinical use of dandelion have been reported, all of which exceeded the normal dose, respectively 20 g, 30 g, and 30 g [170,171,172]. Toxicological safety evaluation is essential for plant application and new drug development. Therefore, further research on toxicity is needed to provide a more reliable basis for medication use.

## 9. Conclusions and Future Perspectives

In this paper, the latest review of *Taraxaci herba* was made through textual research of herbal medicine, traditional medicine, phytochemistry, pharmacology, and artificial cultivation. According to historical documents, *Taraxaci herba* is a plant medicine that can be used as a whole herb, and it has been used for a long time to treat acute inflammation, colds and fever, bacterial infections, and so on. In China, *Taraxaci herba* is often used in combination with other natural medicines, and a large number of medicinal prescriptions for *Taraxaci herba* are recorded.

There are a total of 84 species of *Taraxacum* in China, and 27 of them can be used for medicinal purposes, among which *T. mongolicum*, *T. sinicum*, *T. officinale*, *T. scariosum* and *T. platycarpum* are the most common. Therefore, the research on the chemical components of *Taraxacum* plants mainly focuses on the above five species, and there is a lack of research on other species of *Taraxacum*. The research on the extraction of active components of dandelions lags, and the application of advanced technologies, such as ultrasonic-assisted extraction, microwave-assisted extraction, or supercritical fluid extraction technology, is relatively lacking.

The roots, stems, leaves, and flowers of *Taraxaci herba* are all rich in flavonoids, but the content of flavonoids in different parts is obviously different (flowers > leaves > roots), which are mainly concentrated in the aboveground parts. Terpenoids in *Taraxacum* plants are mainly triterpenoids and sesquiterpenes, among which pentacyclic triterpenoid taraxasterol is one of the main active components in *Taraxacum* plants, and sesquiterpenes are the main source of the bitter taste of *Taraxacum*.

Current phytochemical and pharmacological studies show that *Taraxaci herba* has significant anti-inflammatory, antioxidant, anti-tumor, and antibacterial effects. Pharmacological research provides a scientific theoretical basis for the routine application of *Taraxaci herba* in classical medicine, especially in the treatment of inflammation. *Taraxaci herba* has a remarkable therapeutic effect on mastitis in clinical practice in China, mainly achieved through external application, oral administration, a combination with other natural medicines, and integrated Chinese and Western medicine approaches. Previous pharmacological experiments have confirmed that dandelion has a certain inhibitory effect on the growth and reproduction of cancer cells such as in breast cancer, liver cancer, lung cancer, and gastric cancer. It is basically non-toxic, has few adverse reactions, and can be developed as a potential natural anti-cancer drug or an adjunct for cancer treatment. *Taraxaci herba* is usually used in combination with other natural plants and animal drugs, but there is relatively little research on the synergistic mechanism. Therefore, it seems to be a new way to explore the synergistic mechanism of drugs between specific natural animals, plants, and *Taraxaci herba*. Furthermore, the processing and application methods of dandelion are single, inevitably causing problems such as the loss of active ingredients and low bioavailability. Innovative nano-encapsulation technology and sustained-release dosage forms can effectively improve bioavailability and therapeutic targeting, which is worthy of further research. *Taraxaci herba* plants are widely distributed, and the accumulation of metabolites in *Taraxaci herba* plants is quite different due to different harvesting times and environmental differences between different producing areas. *Taraxaci herba* has broad commercial potential. Its rich medicinal value provides a new research and development direction for the pharmaceutical industry, which is expected to develop a series of efficient and low-toxic natural medicine products. In addition, Taraxaci herba also has huge development space in the fields of food and health care products. For example, it can be made into d*andelion* tea and dandelion health care products. In the cosmetics industry, it can be used as a natural antioxidant and anti-inflammatory ingredient and applied to the development of skin care products. In the medical market, drugs made from its extracts may occupy a certain market share. With people’s preference for natural ingredients, the market demand for natural medicines and health care products based on Taraxaci herba is expected to continue to grow. At the same time, its application value in ecological restoration and environmental governance may also attract investment and development from related enterprises. We hope that the above information will help people better understand the traditional medicinal plant *Taraxaci herba*, establish a perfect yield and quality control system, and provide convenience for the development and application of *Taraxaci herba* in the future.

## Figures and Tables

**Table 1:** A total of 84 species of the genus *Taraxacum* in China.

No.	Latin Name	Distribution Area	Altitude
1	*Taraxacum mongolicum*	Heilongjiang, Jilin, Liaoning, Inner Mongolia, Hebei, Shandong, China; Mongolia; North Korea; Russia	100–1500 m
2	*Taraxacum brassicaefolium*	Heilongjiang, Jilin, Liaoning, Inner Mongolia, Hebei, China;	500–1570 m
3	*Taraxacum coreanum*	Heilongjiang, Jilin, Liaoning, Inner Mongolia, Hebei, China; Russia; North Korea	70–500 m
4	*Taraxacum variegatum*	Heilongjiang, Jilin, Liaoning, Inner Mongolia, Hebei, China;	800–1600 m
5	*Taraxacum scariosum*	Heilongjiang, Jilin, Liaoning, Inner Mongolia, Hebei, Shanxi, Shaanxi, China; Russia; Mongolia	1380–3380 m
6	*Taraxacum borealisinense*	Heilongjiang, Jilin, Liaoning, Inner Mongolia, Hebei, Shanxi, Shaanxi, Gansu, Qinghai, Henan, Sichuan, and Yunnan; China; Mongolia; Russia	300–2900 m
7	*Taraxacum scariosum*	Heilongjiang, Inner Mongolia, Shanxi, Xinjiang, Tibet, China; Kazakhstan; Mongolia; Russia	900–3000 m
8	*Taraxacum apargiaeforme*	Heilongjiang, Jilin, Liaoning, China	50–600 m
9	*Taraxacum lamprolepis*	Heilongjiang, Jilin, Liaoning, Inner Mongolia, China	230–810 m
10	*Taraxacum sinicum*	Heilongjiang, Jilin, Liaoning, Inner Mongolia, China; Mongolia; Russia	120–2900 m
11	*Taraxacum ohwianum*	Heilongjiang, Jilin, Liaoning, China; North Korea, Russian Far East	50–1370 m
12	*Taraxacum erythropodium*	Heilongjiang, Jilin, Liaoning, Inner Mongolia, China	620–4900 m
13	*Taraxacum platypecidum*	Heilongjiang, Jilin, Liaoning, Inner Mongolia, Hebei, Shanxi, Shaanxi, Henan, Hubei, Sichuan, China; North Korea; Russia; Japan	1900–3400 m
14	*Taraxacum antungense*	Heilongjiang, Jilin, Liaoning, China	1200–1400 m
15	*Taraxacum altaicum*	Xinjiang (Urumqi, Qitai, Altay), China; Kazakhstan; Russia	2000–2500 m
16	*Taraxacum alatopetiolum*	Xinjiang (Urumqi), China	3400 m
17	*Taraxacum bessarabicum*	Xinjiang (Urumqi, Yili), China; Mongolia; Kazakhstan; Iran; Parts of Europe	2300–2800 m
18	*Taraxacum bicorne*	Xinjiang, China, Gansu; Kazakhstan; Afghanistan; Pakistan; India; Iran	2050–3300 m
19	*Taraxacum centrasiaticum*	Xinjiang, China	3500 m
20	*Taraxacum compactum*	Xinjiang, China; Kazakhstan	700–1700 m
21	*Taraxacum dealbatum*	Xinjiang, China; Russia; Kazakhstan; Mongolia	1300–4390 m
22	*Taraxacum dissectum*	Xinjiang, China; Russia	3600 m
23	*Taraxacum ecornutum*	Xinjiang (Urumqi, Yining), China; Kazakhstan	600–800 m
24	*Taraxacum erythrospermum*	Xinjiang, China; Kazakhstan	3400 m
25	*Taraxacum glabrum*	Xinjiang (Urumqi, Fukang, Nilek, Heshuo, Luntai, Kuqa, Artux, Yecheng), China; Kazakhstan; Russia	2300–4200 m
26	*Taraxacum goloskokovii*	Xinjiang (Tashkurgan), China; Kazakhstan, Kyrgyzstan	3000–3700 m
27	*Taraxacum kok-saghyz*	Xinjiang, China; Kazakhstan	1100–4050 m
28	*Taraxacum lilacinum*	Xinjiang (Urumqi, Fukang, Brzin), China; Kazakhstan; Kyrgyzstan	2500 m
29	*Taraxacum lipskyi*	Xinjiang (Qinghe, Fuyun), China; Kazakhstan; Kyrgyzstan	3358 m
30	*Taraxacum longipyramidatum*	Xinjiang (Urumqi, Manas, Tacheng), China; Kazakhstan; Kyrgyzstan	1500 m
31	*Taraxacum luridum*	Xinjiang (Tashkurgan), China; Russia; Kazakhstan; Pakistan; Afghanistan; Iran	3000 m
32	*Taraxacum minutilobum*	Xinjiang (Tashkurgan), China; Kazakhstan; Kyrgyzstan, Uzbekistan	3000–3700 m
33	*Taraxacum monochlamydeum*	Xinjiang, Gansu, China; Kazakhstan; Afghanistan; Pakistan; India; Iran	Not applicable
34	*Taraxacum multiscaposum*	Xinjiang (Urumqi, Wusu), China; Kazakhstan; Kyrgyzstan; Afghanistan; Iran	800–1000 m
35	*Taraxacum officinale*	Xinjiang, China; Kazakhstan; Kyrgyzstan; Europe; North America	700–2200 m
36	*Taraxacum pingue*	Xinjiang (Hejing, Tashkurgan), China, Kazakhstan, Kyrgyzstan, Russia	3950–4500 m
37	*Taraxacum potaninii*	Xinjiang, China	2000–2400 m
38	*Taraxacum przevalskii*	Xinjiang, Tibet, China	5000 m
39	*Taraxacum xinyuanicum*	Xinjiang (Xinyuan), China	1500 m
40	*Taraxacum tianschanicum*	Xinjiang (Tianshan), China; Kazakhstan	900–2500 m
41	*Taraxacum sumneviczii*	Xinjiang (Huocheng), China; Kazakhstan; Kyrgyzstan; Russia	1400 m
42	*Taraxacum subglaciale*	Xinjiang (Tashkurgan), China; Kazakhstan	3500–4500 m
43	*Taraxacum stenolobum*	Xinjiang (Qinghe, Altay, Habahe, Brzin), China; Russia; Kazakhstan	3100–3800 m
44	*Taraxacum stanjukoviczii*	Xinjiang (Hotan), China; Kazakhstan; Kyrgyzstan; Iran; Afghanistan	3000–4000 m
45	*Taraxacum sinotianschanicum*	Xinjiang, China	3440 m
46	*Taraxacum roborovskyi*	Xinjiang (East Tianshan), China	5000 m
47	*Taraxacum repandum*	Xinjiang (Aheqi), China; Kazakhstan; Kyrgyzstan	2900 m
48	*Taraxacum qirae*	Xinjiang (Cele), China	3000 m
49	*Taraxacum pseudoroseum*	Xinjiang (Urumqi, Fukang, Shawan, Qitai, Altay, Yining, Nilek), China; Kazakhstan	2500–3300 m
50	*Taraxacum pseudominutilobum*	Xinjiang (Tashkurgan, Artux), China; Kazakhstan; Uzbekistan	3000–3700 m
51	*Taraxacum pseudoatratum*	Xinjiang (Tacheng, Miquan, Balikun, Tex, Zhaosu and Jingjing), China; Kazakhstan; Russia	3700–5000 m
52	*Taraxacum pseudoalpinum*	Xinjiang (Qinghe and Xinyuan), China; Kazakhstan; Kyrgyzstan	900–1300 m
53	*Taraxacum abax Kirschner*	Xinjiang, Hebei, China	1500–1600 m
54	*Taraxacum apargiaeforme*	Tibet, Sichuan (Lixian, Wenchuan, Marcand), China	3000–3800 m
55	*Taraxacum brevirostre*	Tibet, Gansu, Qinghai, China; Afghanistan; Pakistan; Iraq; Iran; Turkey	1700–5000 m
56	*Taraxacum calanthodium*	Tibet, Shaanxi, Gansu, Qinghai, Sichuan, China	2500–4300 m
57	*Taraxacum eriopodum*	Tibet, Gansu, Qinghai, Yunnan (Lijiang, Zhongdian and Yongsheng), Sichuan, China; Sikkim; Bhutan; Nepal; India	3000–5300 m
58	*Taraxacum forrestii*	Tibet, Yunnan, China; India	4200 m
59	*Taraxacum glaucophyllum*	Tibet, Qinghai, Sichuan, Yunnan, China	2800–4300 m
60	*Taraxacum grypodon*	Tibet, Sichuan, China	1430–4200 m
61	*Taraxacum lanigerum*	Tibet, Qinghai, China; Nepal	3900–4600 m
62	*Taraxacum albiflos*	Tibet, Gansu, Qinghai, Xinjiang, China; India; Iran; Pakistan; Russia	2500–6000 m
63	*Taraxacum ludlowii*	Tibet (Lhasa, Dangxiong, Linzhou); China	3900–5300 m
64	*Taraxacum lugubre*	Tibet, Gansu, Qinghai, Sichuan, China.	2800–4200 m
65	*Taraxacum maurocarpum*	Tibet, Qinghai, Sichuan (Ganzi, Aba), China; Russia; Iran; Afghanistan; Pakistan	3000–4500 m
66	*Taraxorum mitalii*	Tibet, China; Bhutan; Sikkim; Nepal	2400–4500 m
67	*Taraxacum parvulum*	Tibet, Shanxi, Qinghai, Sichuan, Yunnan, China; Bhutan, India, Pakistan	1500–4500 m
68	*Taraxacum tibetanum*	Tibet, Qinghai, Sichuan (Ganzi, Aba), Yunnan, China; Sikkim; Bhutan	3600–5300 m
69	*Taraxacum subcoronatum*	Tibet, China	4500 m
70	*Taraxacum sikkimense*	Tibet, Qinghai, Sichuan, Yunnan (Lijiang, Zhongdian), China; Sikkim; Nepal; Pakistan	2800–4800 m
71	*Taraxacum sherriffii*	Tibet, Qinghai, Yunnan, China; Kashmir	2300–4500 m
72	*Taraxacum roseoflavescens*	Tibet, China	4300 m
73	*Taraxacum stenoceras*	Tibet, Gansu, Qinghai, Sichuan, China.	3000–4500 m
74	*Taraxacum sinomongolicum*	Beijing, Inner Mongolia, Hebei, China	512 m
75	*Taraxacum chionophilum*	Sichuan (Lixian, Marcand, Songpan), China	2700–4600 m
76	*Taraxacum indicum*	Sichuan, Yunnan, China; India; Viet Nam	1300–3800 m
77	*Taraxacum kozlovii*	Gansu, China	2300 m
78	*Taraxacum pseudostenoceras*	Gansu (Xiahe), Qinghai (Zeku, Tongren, Guide), China; Nepal	2300–3500 m
79	*Taraxacum suberiopodum*	Yunnan (Zhongdian, Ninglang), China	3100–3400 m
80	*Taraxacum dasypodum*	Yunnan (Lijiang, Deqin, Zhongdian, Jingdong), China	1900–3200 m
81	*Taraxacum duplex*	Shandong; China	40–200 m
82	*Taraxacum platypecidum*	Shanxi, China	2200 m
83	*Taraxacum nutans*	Shanxi, Ningxia (Haiyuan), Hebei (Fuping); China	1100–3200 m
84	*Taraxacum scariosum*	Heilongjiang, Jilin, Liaoning, Inner Mongolia, Hebei, China; Russia	70–1750 m

Not applicable means that no relevant information is to be found.

**Table 2:** Traditional Prescription Drugs Related to *Taraxaci herba* in China.

Traditional Uses	Major Component	Formulation	Ref.
Acute mastitis	Pugongying, Baijili	Apply externally	Bencao Gangmu (Ming Dynasty, AD 1578–1596) [26]
Acute mastitis	Pugongying, Rendong, Gancao	Decoction	Secret record of surgery (Qing Dynasty, AD 1694) [27]
Acute mastitis	Pugongying, Xiangfu	Decoction	Selected materials of new medical treatment of Chinese herbal medicine [28]
Scrofula, Phlegm nucleus	Pugongying, Xiangfu, Yangtigen, Dajin, Shancigu, Huzhangcao	Decoction	Diannan Bencao (Ming Dynasty, AD 1436) [29]
Accumulates milk	Pugongying	Apply externally	Meishi Prescription (Song Dynasty, AD 785–820) [30]
Acute suppurative infection	Pugongying, Ruxiang, Moyao, Gancao	Decoction	National Chinese herbal medicine compilation [31]
Hepatitis, cholecystitis	Pugongying, Yinchenhao, Chaihu, Zhizi, Yujin, Fuling	Decoction	Commonly used Chinese herbal medicines in Nanjing [32]
Chronic gastritis, Gastric ulcer	Pugongying, Diyu, Baiji	Pulverization	Commonly used Chinese herbal medicines in Nanjing [32]
Carbuncle of lung, Acute appendicitis, Suppurative infection of back	Pugongying, Jinyinhua, Xuanshen, Danggui	Decoction	Secret record of surgery (Qing Dynasty, AD 1694) [33]
Acute conjunctivitis	Pugongying, Juhua, Bohe, Cheqianzi	Decoction	Anhui Chinese herbal medicine [34]
Defecate haemorrhage	Pugongying, Huaijiaozi, Shibing, Heimuer, Shenqu	Decoction	He Shi prescription (Qing Dynasty, AD 1672) [35]
Urethritis	Pugongying, Cheqiancao, Qumai, Rendongteng, Shiwei	Decoction	Handbook of Chinese Herbal Medicine in Qingdao [36]
Fixing teeth	Pugongying, Qingyan, Shiyan, Huaijiaozi	Apply externally	He Shi prescription (Qing Dynasty, AD 1672) [35]
Acute icteric hepatitis	Pugongying, Yinchenhao, Tufuling, Baimaogen, Diercao	Decoction	Herbology of Changbai Mountain [37]
Acute biliary tract infection	Pugongying, Zhencicao, Haijinsha, Lianqiancao, Yujin, Chuanlianzi	Decoction	National Chinese herbal medicine compilation [31]
Acute appendicitis	Pugongying, Diercao, Banbianlian, Zelan, Qingmuxiang	Decoction	National Chinese herbal medicine compilation [31]

**Table 3:** Main Flavonoids in *Taraxaci herba*.

No.	Names	Extract Source	Molecular Formula	CAS	Refs.
**1**	Luteolin	*T. Mongolicum, T. Sinicum, T. officinale*	C_15_H_10_O_6_	491-70-3	[42,43,47]
**2**	Quercetin	*T. Mongolicum, T. Sinicum, T. officinale*	C_15_H_10_O_7_	117-39-5	
**3**	Quercetin-7-O-β-D-glucoside	*T. Mongolicum, T. officinale*	C_21_H_20_O_12_	491-50-9	
**4**	Luteolin 7-rutcoside	*T. Mongolicum, T. officinale, T. falcilobum*	C_27_H_30_O_15_	20633-84-5	
**5**	lsorhamnetin 3,7-0-diglucoside	*T. Mongolicum, T. officinale*	C_28_H_32_O_17_	6758-51-6	
**6**	Isoquercitrin	*T. Mongolicum, T. officinale*	C_21_H_20_O_12_	482-35-9	[42,43,47,48]
**7**	Diosmetin	*T. Mongolicum, T. officinale*	C_16_H_12_O_6_	520-34-3	
**8**	Luteolin-7-O-β-D-glucoside	*T. Mongolicum, T. officinale*	C_21_H_20_O_11_	5373-11-5	
**9**	Hesperidin	*T. Mongolicum, T. officinale, T. Sinicum*	C_28_H_34_O_15_	520-26-3	
**10**	Apigenin 7-glucoside	*T. Mongolicum, T. Sinicum, T. officinale*	C_21_H_20_O_10_	578-74-5	[47,49,50]
**11**	Apigenin	*T. Mongolicum, T. Sinicum, T. officinale*	C_15_H_10_O_5_	520-36-5	
**12**	Rutin	*T. Mongolicum, T. Sinicum, T. officinale*	C_27_H3_0_O_16_	153-18-4	
**13**	Calquiquelignan E	*T. Mongolicum, T. Sinicum*	C_26_H_24_O_10_	1292294-31-5	[39,51]
**14**	Calquiquelignan D	*T. Mongolicum, T. Sinicum*	C_26_H_24_O_10_	1928715-38-1	
**15**	Genistin	*T. Mongolicum, T. Sinicum*	C_21_H_20_O_10_	529-59-9	
**16**	Chrysoeriol	*T. Mongolicum, T. Sinicum*	C_16_H_12_O_6_	491-71-4	
**17**	Isorhamnetin	*T. Mongolicum, T. Sinicum*	C_16_H_12_O_7_	480-19-3	
**18**	Isorhamnetin-3-O-glucoside	*T. officinale*	C_22_H_22_O_12_	6743-92-6	
**19**	Hesperetin 7-O-β-D-glucuronide	*T. mongolicum*	C_22_H_22_O_12_	1237479-09-2	[45]
**20**	Hesperetin 5-O-glucoside	*T. mongolicum*	C_22_H_24_O_11_	69651-80-5	

**Table 4:** Major Terpenoids and Steroids in *Taraxaci herba*.

No.	Names	Extract Source	Molecular Formula	CAS	Refs.
**1**	Psi-taraxasterol acetate	*T. mongolicum, T. officinale, T. falcilobum*	C_32_H_52_O_2_	4586-65-6	[54]
**2**	Paradiol	*T. mongolicum, T. officinale*	C_30_H_50_O_2_	20554-95-4	[9]
**3**	Taraxasterol	*T. mongolicum, T. officinale*	C_30_H_50_O	1059-14-9	
**4**	Psi-taraxasterol	*T. mongolicum, T. officinale*	C_30_H_50_O	464-98-2	
**5**	Arnidiol	*T. Mongolicum, T. officinale*	C_30_H_50_O_2_	6750-30-7	
**6**	Taraxasteryl acetate	*T. mongolicum, T. officinale*	C_32_H_52_0_2_	6426-43-3	[44,57]
**7**	Artemisinin	*T. mongolicum, T. officinale*	C_15_H_22_O_5_	63968-64-9	
**8**	α-Amyrin	*T. mongolicum, T. officinale*	C_30_H_50_O	5937-48-4	[58,59]
**9**	β-Amyrin acetate	*T. mongolicum, T. officinale*	C_32_H_52_O_2_	1616-93-9	
**10**	α-Amyrin acetate	*T. mongolicum, T. officinale*	C_32_H_52_O_2_	863-76-3	
**11**	β-Sitosterol	*T. mongolicum, T. officinale, T. antungense*	C_29_H_50_O	83-46-5	[50,60]
**12**	Lupeol	*T. mongolicum, T. officinale T. antungense*	C_30_H_50_O	545-47-1	
**13**	Stigmasterol	*T. mongolicum, T. officinale T. antungense*	C_29_H_48_O	83-48-7	
**14**	Cryptomeridiol	*T. mongolicum, T. officinale*	C_15_H_28_O_2_	4666-84-6	[61]
**15**	Campesterol	*T. mongolicum, T. officinale*	C_28_H_48_O	474-62-4	[56,62]
**16**	Daucosterol	*T. mongolicum, T. officinale*	C_35_H_60_O_6_	474-58-8	[50,59,63]
**17**	Germacranolide sonchuside A	*T. mongolicum, T. officinale*	C_21_H_32_O_8_	111618-82-7	
**18**	β-Amyrin	*T. mongolicum, T. officinale*	C_30_H_50_O	559-70-6	
**19**	sesquiterpene lactone	*T. officinale, T. obovatum*	C_34_H_50_O_12_	67526-95-8	[55]
**20**	Sonchuside A	*T. officinale, T. obovatum*	C_21_H_32_O_8_	111618-82-7	

**Table 5:** Phenolic acids in *Taraxaci herba*.

No.	Names	Extract Source	Molecular Formula	CAS	Refs.
**1**	Chlorogenic acid	*T. mongolicum, T. officinale, T. falcilobum, T. siphonanthum*	C_16_H_18_O_9_	327-97-9	[43,65]
**2**	Chicoric Acid	*T. mongolicum, T. officinale*	C_22_H_18_O_12_	6537-80-0	
**3**	Caffeic acid	*T. mongolicum, T. officinale, T. falcilobum, T. siphonanthum*	C_9_H_8_O_4_	331-39-5	
**4**	4-Hydroxybenzoic acid	*T. mongolicum, T. officinale*	C_7_H_6_O_3_	99-96-7	
**5**	Vanillic acid	*T. mongolicum, T. officinale*	C_8_H_8_O_4_	121-34-6	[49,69]
**6**	Protocatechuic acid	*T. mongolicum T. officinale*	C_7_H_6_O_4_	99-50-3	
**7**	3,5-Dihydroxybenzoic acid	*T. mongolicum T. officinale*	C_7_H_6_O_4_	99-10-5	
**8**	3,5-di-O-caffeoylquinic acid	*T. mongolicum T. officinale*	C_25_H_24_O_12_	89919-62-0	
**9**	Isochlorogenic acid B	*T. mongolicum T. officinale*	C_25_H_24_O_12_	14534-61-3	
**10**	Ferulic acid	*T. mongolicum, T. officinale*	C_10_H_10_O_4_	331-39-5	[61,66]
**11**	Gallic acid	*T. mongolicum, T. officinale*	C_7_H_6_O_5_	149-91-7	
**12**	Caftaric acid	*T. mongolicum, T. officinale*	C_13_H_12_O_9_	67879-58-7	
**13**	Methyl caffeate acid	*T. mongolicum*	C_10_H_10_O_4_	3843-74-1	[68]
**14**	Ethyl 2-(4-hydroxyphenyl) acetate	*T. mongolicum*	C_10_H_12_O_3_	17138-28-2	
**15**	Methyl 4-Hydroxyphenylacetate	*T. mongolicum*	C_9_H_10_O_3_	22446-37-3	
**16**	Phenylacetic acid	*T. mongolicum*	C_8_H_8_O_2_	103-82-2	
**17**	Vanillin	*T. mongolicum, T. officinale*	C_8_H_8_O_3_	121-33-5	[70]
**18**	Coniferaldehyde	*T. mongolicum, T. officinale*	C_10_H_10_O_3_	458-36-6	
**19**	P-methoxyphenylglyoxylic acid	*T. mongolicum, T. officinale*	C_9_H_8_O_4_	7099-91-4	
**20**	Isovanillin	*T. mongolicum, T. officinale*	C_8_H_8_O_3_	86884-84-6	
**21**	Butyl rosmarinate	*T. mongolicum*	C_22_H_24_O_8_	222713-83-9	[63]
**22**	3,4-Di-O-caffeoylquinic acid methyl ester	*T. mongolicum*	C_26_H_26_O_12_	114637-83-1	
**23**	Butylchlorogenate	*T. mongolicum*	C_20_H_26_O_9_	132741-56-1	

**Table 6:** Environmental factors that influence *Taraxaci herba*.

Influencing Factor	Research Objects	Summary
Illumination intensity	Plant growth; Active ingredient content	Under natural light: The contents of choline and flavonoids were the highest at 80% transmittance. The content of triterpenoids was the highest under full light. *Taraxaci herba* with light transmittance of 60~80% has the strongest antioxidant capacity in vitro; 60% light transmittance is beneficial to the growth of *Taraxaci herba* leaves, and more than 60% light transmittance inhibits the growth of its aboveground parts [154,155,156].
Fertilizer use; Cultivation season	Yield and quality	Planting should be carried out in spring and autumn. Proper application of nitrogen fertilizer can improve the yield and nutritional quality of *Taraxaci herba* [157].
Reserve temperature	Physiological metabolism	*Taraxaci herba* stored at 2 °C has the highest active ingredient content, MDA content, and high SOD activity, suggesting that storage at 2 °C significantly delays its aging process and prolongs its storage life [158,159].
Soil environment	Plant growth; Active ingredient content	The contents of flavonoids, saponins, and chlorogenic acid in *Taraxaci herba* leaves were increased by low salt, alkali stress, and moderate alkali stress. High salt stress (≥0.2% NaCl) significantly inhibited the growth of leaves and roots of *Taraxaci herba* and significantly reduced the content of cichoric acid. Arsenic inhibits the synthesis of chlorogenic acid in *Taraxaci herba*, and elements such as calcium and zinc are inversely related to the contents of chlorogenic acid and caffeic acid in *Taraxaci herba*. The contents of phenolic acids (chlorogenic acid, caffeic acid, ferulic acid) and flavonoids (rutin, luteolin, quercetin) in *Taraxaci herba* grass in a soil environment are higher than those in the wet environment [154,160,161,162].

## Data Availability

No data were used for the research described in the article.

## Glossary

TS	Taraxasterol	TDS	Taraxaci herba polysaccharides
TF	Taraxaci herba flavone	Bcl2	B-cell lymphoma-2
STAT	Signal transducer and activator of transcription	JAK	Janus kinase
JNK	c-Jun N-terminal kinase	SOCS3	Cytokine signaling pathway inhibitor 3
APAP	Acetaminophen	HO-1	Heme Oxygenase-1
Nrf2	Nuclearrespiratoty factor 2	PGE2	Prostaglandin E2
Hint1	Histidine triad nucleotide-binding protein 1	IL-1	Interleukin-1
IL-6	Interleukin-6	NF-κB	Nuclear factor-κB
MAPK	Mitogen-activated protein kinase	TIMP	Tissue inhibitor of matrix metalloproteinases
Cox-2	Cyclooxygenase-2	TNF-α	Tumor necrosis factor alpha
IL-1β	Interleukin—1β	MPO	Myeloperoxidase
SOD	Superoxide dismutase	GSH	Glutathione
MDA	Malondialdehyde	MAO	Monoamine oxidase
LPF	Leukocytosis promoting factor	ROS	Reactive oxygen species
MMP	Matrix metallopeptidase	CAT	Catalase
MIC	Minimum inhibitory concentration	TNBC	Triple negative breast cancer
IL-10	Interleukin-10	ERK	Extracellular signal-regulated kinase
MAPK	Activated protein kinase	BAX	Bcl2 Associated x protein
